# Assessing the impact of health research on health policies: a study of the Dodowa Health Research Centre, Ghana

**DOI:** 10.1186/s12913-017-2383-0

**Published:** 2017-06-24

**Authors:** Blanca Escribano-Ferrer, Jayne Webster, Margaret Gyapong

**Affiliations:** 1 Faculty of Infectious and Tropical Diseases, London School of Hygiene and Tropical Medicine, London, UK; 2 Dodowa Health Research Centre, Ghana Health Service, Dodowa, Ghana

**Keywords:** Research policy impact, Research translation into policies, Research process, Policy analysis

## Abstract

**Background:**

The importance of assessing research impact is increasingly recognised. Ghana has a long tradition of research dating from the 1970s. In the Ghana Health Service there are three health research centres under the Research and Development Division. Dodowa Health Research Centre (DHRC) is the youngest in the country dating from the 1990s. The objective of this study is to analyse the influence of the research conducted in DHRC on national and local health policies.

**Methods:**

The study used the Research Impact Framework. Six projects were selected based on a set of criteria. Thirteen interviews were conducted with researchers and policy makers using a semi-structured interview guide.

**Results:**

DHRC had numerous policy impacts in terms of researchers participating in policy networks, increasing political capital and influencing policy documents. Factors identified to be associated with policy impact included collaboration with policy makers at the design stage, addressing health priorities, and communicating results mainly through the participation in annual review meetings.

**Conclusions:**

DHRC was successful in influencing health policies. Recommendations were made that could be included in the DHRC strategic planning to improve the research process and its policy impact.

**Electronic supplementary material:**

The online version of this article (doi:10.1186/s12913-017-2383-0) contains supplementary material, which is available to authorized users.

## Background

Research impact, understood as the benefits from research or the payback of research [1–3], is increasingly becoming recognised as important. Public and private funders want to know the value received for the funds given, and want to see tangible results in order to justify continuing, or increasing funds and support for research [4–6].

Since the mid-1990s, efforts were made to evaluate research impact and justify expenditure on health services research using several approaches. The traditional approach focussed on academic production such as the number of publications [3, 7, 8], as well as in assessing the economic value of the research [9] and the health impact in terms of mortality reduction [10]. Since 1996 new methodologies have been developed combining several categories or areas where research can have an impact. Each of these new methodologies has a different focus. For example, one methodology considers a logic model, that starts with research conception and ends with results dissemination and utilization [5]. Another approach applies a systems perspective, with the objective to strengthen the health research systems [11]. Others focus on informing decision making and effectively transmitting evidence to policymakers [12]; developing a set of impact as well as performance indicators [13, 14]; making a practical approach, trying to identify categories that have not yet been taken into account [15]; addressing interdisciplinary research [16] and lastly, taking into account small-scale projects [17].

These different methodologies have been developed progressively in an attempt to address important aspects related to research impact: (i) the length of time required for research to have an effect; (ii) the need for considering different areas of effect and different types of research; (iii) how to address difficulties in quantifying research impacts; (iv) how to address difficulties in attributing a policy or an impact to a particular research result; and finally (v) the need for a method to be easy to use and (vi) to allow comparisons with regards to time and within institutions.

Ghana has a long tradition in medical research dating from the late 1970s and 1980s. In the Ghana Health Service and under the Research and Development Division there are 3 health research centres that represent the three ecological zones in the country: Navrongo Health Research Centre (NHRC) in the north, Kintampo Health Research Centre (KHRC) in the centre of the country, and Dodowa Health Research Centre (DHRC) in the south. DHRC is the youngest in the country established in the early 1990s [18, 19].

DHRC is situated in Dodowa, the district capital of the Dangme West district (now Shai-Osudoku and Ningo-Prampram districts since July 2012) of the Greater Accra Region. At the time of the study, the population of Dangme West district was 148,909 inhabitants with 21 static health facilities (15 public and 6 private), 53 chemical sellers, 5 Pharmacies and 5 laboratories (3 public and 2 private) [20]. In coordination with the national level, the district health directorate prepared annual plans and annual reports- the later presented during annual review meetings. DHRC was created as part of the agreements between the Government of Ghana and the British Oversees Development Agency (now DFID) to establish an operational research satellite station in the early 1990’s. Since its origin, DHRC has developed a close relationship with the Dangme West District Health administration. The first building was placed in the district health administration, and its staff join the health district annual reviews meetings.

The research centre has been growing since its origin in terms of human and financial resources, equipment, research projects and influence in the health sector. But the limited resources and unlimited needs of the Ghana Health Sector require that the research centre remains effective, in terms of being able to show the impact of its research.

In 2005, the research centre set up a Health and Demographic Surveillance System (HDSS). This HDSS covers an area of 1528.9 km^2^. In terms of research, DHRC has focused on malaria operational research since its inception. Due to the expansion of the centre, a new infrastructure was built in 2006/2007 to host the new research centre [19]. From 2005 to 2011, the annual budget of the centre increased from US$ 371,000$ to 897,000$ and the staff boost from 33 to 120 people, being 7 in administration, 7 in support services, 17 in computer centre, 69 field workers and 20 scientists. The number of publications has been low, ranging from 0 to 4 papers published per year for the period 2004–2011. In 2011, DHRC developed its first strategic plan 2012–2016 to guide the continuous growth of the research centre, to improve its performance and to be more efficient [20]. This strategic plan includes the mission, vision and values of the health research centre, the strategic goals, its research agenda, a chronogram, a budget and a monitoring and evaluation plan. A set of indicators was developed to be monitored annually. Some of these indicators are the number of peer reviewed publications, the number of posters presented, number of presentations of research results to policy makers at national level, number of partnerships and collaborations with other institutions, website with actualized information and annual reports disseminated to funders, the Ghana Health Service and the Ministry of Health. However, these indicators cannot inform about the use of the evidence generated for policy making. This study aims to analyse the influence of the research conducted in DHRC on national and local health policies. It will complement the monitoring and evaluation plan, and its findings might be included in the next strategic plan. This is the first study addressing research impact on health policies conducted in any of the three health research centres.

## Methods

### Analytical framework: Research impact framework

Among the different approaches to assess research impact mentioned above we chose the Research Impact Framework (RIF) for this study. The Research Impact Framework (RIF) was developed by Kuruvilla et al. [15, 21] as a relatively economic way to identify the academic and ‘real world’ impacts of research projects and programs. The RIF is appropriate for this study because it focuses on policy impact, is potentially easy to implement, allows identification of unexpected impacts and facilitates comparison across time and cases. The RIF helps to systematically analyse areas where projects could have an impact. It describes 4 categories that can be influenced by research: research related impact, policy impact, service impact and societal impact (Table 1). Each category has subcategories that can be assessed for possible impact of a specific research project. Policy impact refers to research informing and influencing policy [15]. The subcategories to explore the policy impact are participation in policy networks, the increase in political capital, the level of policy-making influenced, the type of policies influenced and the nature of the policy impact.

**Table 1:** Research Impact Framework [15]

Research-related impacts	Policy impacts	Service impacts	Societal impacts
• Type of problem/knowledge • Research methods • Publications and papers • Products, patents and translatability potential • Research networks • Leadership and awards • Research management • Communication	• Level of policy-making • Type of policy • Nature of policy impact • Policy networks • Political capital	• Type of services: health/intersectoral • Evidence-based practice • Quality of care • Information systems • Services management • Cost-containment and cost-effectiveness	• Knowledge, attitudes and behaviour • Health literacy • Health status • Equity and human rights • Macroeconomic/related to the economy • Social capital and empowerment • Culture and art • Sustainable development outcomes

To use this framework, [21] proposed an approach that includes project selection, in-depth interviews, documentary analysis and the elaboration of impact narratives for each project.

### Research projects selection

Research projects conducted in Dodowa were selected to assess their policy impact based on the following criteria:

(i) Projects had to be completed between 2004 and 2010 to allow time for the generation of the evidence and for the findings to have had an impact. The lower limit of 2004 was set because of records availability and to avoid recall bias.

(ii) Projects needed to reflect a broad range of research conducted in Dodowa. They reflected a multidisciplinary research, including projects that were focused on clinical practice, on the health system and on the social and behavioural level. They also reflected different types of study design, ranging from qualitative studies to randomized control trials. The grade of difficulty for translating some research results into policies was also considered. Finally, the budget of the project was explored and compared. In practical terms, projects where the researcher moved away from the country or was not available were excluded.

Nine out of eleven projects met the first requirement [18, 19]. From the nine studies, six were selected based on the second requirement and the availability of the researcher-the principal researcher was not available in three of the nine projects. The topic areas of the studies selected were deployment of rectal artesunate, malaria rapid diagnostic tests (RDT), home management of fevers in children under-five, male involvement in family planning (FP) decision making and practice, tuberculosis (TB) enablers’ package and mutual health organizations in Ghana.

### In-depth interviews and documentary analysis

To analyse the influence of the research conducted in DHRC, interviews were conducted using a semi-structured guide based on the RIF to address the policy impact of a specific project. Questions regarding the research process and factors that influence the translation of research into policies were also included. Interviews were conducted with researchers and policy makers related to the research topics. With researchers because they were the more knowledgeable on project details, project implementation, research products and dissemination. With policy makers to compare their knowledge and perceptions with those of the researchers on a specific project. To select the researchers to be interviewed, we identified principal researchers of the selected research projects. To select the policy makers to be interviewed, we identified program coordinators, for example the director of malaria or tuberculosis program if the research project was related to malaria or tuberculosis.

In addition and as suggested by the DHRC Institutional Review Board, policy makers with an important role in research and policy making (even if they were not related to the studies) were also interviewed about the relevance of the projects conducted in Dodowa (addressing real health needs), the research process and factors that influence the translation of research into policies. Examples of policy makers with an important role in research and policy making were the director of Research at the Ministry of Health or the director of Planning at the Ghana Health Service.

In total, 13 interviews were conducted: four with principal researchers, four with policy makers related to projects and five with policy makers not related to the projects under study. Some researchers and some policy makers were involved in more than one study. All interviews were recorded.

A documentary analysis was conducted before and after the interviews. Before the interviews, documentation on the six projects selected were reviewed [18, 19, 22–26]. After the interviews, and because interviewees were asked to show evidence (policy documents) regarding research influencing policy making, a documentary analysis was conducted to verify the inclusion of research findings in those documents. In addition, reported participation of the researchers involved in the selected studies in different committees or networks with policy makers was also verified. Therefore, the documentary analysis conducted after the interviews intended to triangulate results obtained from the interviews.

### Analysis

The analysis of the interviews was done manually. Responses of researchers and policy makers were organised in a table based on themes pre-determined before the interviews, according to the RIF, the research process and categories of factors that might influence the translation of research into policies. Using this classification, general perceptions of researchers and policy makers on themes described above were examined in turn and compared. Then, a review of the policy documents suggested by the participants during the interview was done to verify the inclusion of research findings in national and local health policies. Because these policy documents did not include a bibliography (in most of the cases), it was not possible to ensure that the policies were due/referred to a specific research study. Therefore, it was only possible to state if research findings were present in policy documents.

### Structured narratives

Results of this analysis were presented in project structured narratives**.** This key information includes research objectives, study conception and design, budget, collaborators, main findings, research products, dissemination and policy impact. These narratives were shown to the interviewees to validate that the information given was well reflected in the narratives and to get authorization for their use (Table 2 and Additional file 1: Table S1-S5).

**Table 2:** Deployment of rectal artesunate in the Dangme West district for severe malaria in children under- five

Analysis areas	Key topics	Key dates
Research project focus and funding	Research problem: the project aims to assess the feasibility of the deployment of rectal artesunate at community level.	2004
	Geopolitical context: Ghana, Tanzania and Mozambique.	
	Funders and funding process: WHO through a call process.	
	Budget: 258.000$	
Research project evolution/process	Conception: WHO put a call on the website. Justification: Studies on the efficacy of rectal articulate were conducted in Ghana in Navrongo research centre. Dodowa conducted the only study in Ghana to look at the feasibility of deployment rectal artesunate in a real context. The study was considered by the researcher and by the policy makers as addressing an important health problem, an urgent matter and a complex issue.	
	Study design/Research methods: Observational study with 2 phases (formative and intervention). The design was defined as good quality study.	
	Research collaborators: WHO, Tanzania and Mozambique research centres, malaria control program, district health director and health centres. Those collaborators were involved in the study from the conception until the dissemination of results giving financial and technical support.	
	Key projects events/concerns: turnover of staff already trained and difficulties with following systematically all procedures (at community or health facility) were challenges during the implementation of the study.	
	Main findings/recommendations: the study produced evidence on the feasibility of administrating artesunate at community level, and the compliance of referral to health facility after the drug administration. Results are considered to be clear and concrete.	
	Dissemination of findings: results were presented to the community, to the district authorities, at the regional health management review, at the national dissemination forum, at the 6th INDEPTH scientific in Burkina Faso (“Using community members to dispense rectal artesunate for the initial management of severe malaria in under-five children in a rural district in Ghana”) and at the Global Health Forum in Geneva in 2008 (“Reaching the Un-Reached in the Event of Severe Malaria in Under Five Children in a Rural District in Ghana”). PI believes that the results have been communicated effectively. More than 400 hundred people received the research results.	
Main research products	Project report and Power point presentation: Done. No policy brief.	2006
	Articles: Article published in 2016 [33].	2016
Policy impacts	Level of policy making: the project had an impact at national level, at health managers and at health providers’ level.	
	Type of policy: the study influenced clinical practice policies on the management of malaria cases.	
	Nature of policy impact: This was a mobilization of support where research findings supported the feasibility of including rectal articulate on guides and protocols in Ghana.	
	Policy networks: researchers informed policy makers through the dissemination mechanisms (district, regional and national dissemination forum).	
	Political capital: the researcher believes they gained value in reaching policy agreements. Research results were considered in policy documents. The researcher expressed that the more research is conducted, the more influence researchers gained.	
	Inclusion in policy documents: Recommendations are included in the Anti- Malaria Drug Policy (MoH), Guidelines for Case Management of Malaria and the Home management of Malaria, ARI, and Diarrhoea guidelines Who benefited: all children in Ghana and health managers through capacity building. Unintended outcomes: None.	2007 2009 2010

## Results

Results showed that DHRC had numerous policy impacts (Table 3). The participation of researchers in policy networks is a way to influence policy makers by sharing the researcher’s expertise and results. All selected studies had team members joining policy networks. All of them used the existing country dissemination mechanism involving policy makers, mainly at local and at national level. The regional level was only used in one study.

**Table 3:** Policy impact of research conducted in DHRC. Comparison between studies

Studies selected	Budget	Type of research	Conception/support and collaboration	Dissemination	Policy impact (level, type, nature, policy networks, political capital and references in policy documents)
Deployment of rectal artesunate in the Dangme West district for severe malaria in children under five (2005)	258,000$	Behavioural research*	WHO call. Malaria program (NMCP), Tanzania and Mozambique research centres, district health team and community. Policy makers involved since the beginning.	Power point at local, regional and national and international level. Formal and informal communications. No policy brief. 1 article (not published at the time of our study).	Level: national. Type: clinical practice policies. Nature: supportive way. Policy networks: Participation on the existing dissemination mechanism. Political capital: researcher involved in a technical committee for Home Management of Malaria. Research results can be found in 3 documents [34–36].
Individually randomized trial of rapid diagnostic tests in rural Ghana (2007)	170,500$	Clinical research	PI initiative. GMP and ACT funds. NMCP, LSHTM, and district health team and ACT consortium. Policy makers involved since the beginning.	Power point at local, regional and international level. Report to the NMCP. No policy brief. 4 articles.	Level: National and international. Type: clinical practice policies. Nature: supportive way. Policy networks: Participation on the existing dissemination mechanism. Political capital: researcher involved in task force for malaria diagnosis in Ghana and in the ACT consortium. Research results can be found in 2 documents [35, 37].
Home management of fevers (malaria and pneumonia) in children under-five: a cluster randomized controlled trial in southern Ghana (2007)	856,000$	Clinical research	WHO call. Makerere, Amsterdam and Maastricht Univ., NMCP, Child Health program and district health team. Policy makers involved since the beginning.	Power point to local and International level. No policy brief. 2 articles.	Level: national and international. Type: Service and clinical practice policies. Nature: supportive way. Policy networks: links with INDEPTH, TDR and iCCM. Political capital: mainly at international level. Findings support the home based care strategy and the Malaria, ARI and Diarrhoea Home based care guidelines [36].
Assessments of male involvement in family planning decision making and practice and its influence on the uptake of Family Planning in the Dangme West district (2005)	5400$	Behavioural research	Ghana–Dutch collaboration. District health team involved since the beginning	Power point at local and national level. No Policy brief. No article.	Level: local. Type: service policies. Nature: redefining/widening accepted believes on FP. Policy networks: participation on the existing dissemination mechanism. Political capital: none. District annual reports reflect an increase of community sensitization campaigns. Indicators showed increased on FP acceptors: from 8.7 in 2007 to 47% in 2011 [38–42].
Examination of the TB Enablers Package in the Dodowa sub-district of the Dangme West District in the Greater Accra Region of Ghana (2009)	6000$	Health system research	Georgetown University. District health team involved since the beginning.	Power point at local level. No Policy brief. No article. Informal communication at national level.	Level: local. Type: governance policies. Nature: redefining/widening practices related to TB enablers. Policy networks: participation on the dissemination mechanism at local level. Political capital: increased links with TB program coordinator (new research came to the centre in 2011). District annual report in 2011 showed more detailed description on who benefitted from TB enablers [42].
Mutual health Organizations (MHO’s) in Ghana and implications for improving the success of health Insurance in Ghana (2004)	6081$	Health system research	Ghana–Dutch collaboration. Erasmus Univ., GHS and District health team, involved since the beginning.	Power point at national and international level. Policy brief to GHS. 1 article.	Level: National and international. Type: governance policies. Nature: instrumental use of research designing and implementing the NHIS. Policy networks: participation on the dissemination mechanism at national level. Participation in a team for the NHIS implementation. Political capital: authors are reference people on insurance in Ghana. No policy documents were found that reflect research findings.

Increasing political capital means that researchers gain value and credibility in the dialogue with policy makers, thus being able to influence policies. Authors from four projects were involved in the technical committees to develop guidelines and policy documents (3 at national level and 1 at the international level). These authors are recognised scientists in malaria diagnostics, community management of fevers and the NHIS in Ghana.

Verifying if research results are present in policy documents is a way to check if policies are evidence based. Findings from three of the six projects selected (the *rectal artesunate, the RDT and the home management of fevers projects*) were found in policy documents. Recommendations from two studies were found in district health annual reports (*male involvement in FP and TB enablers projects*). With regards to the mutual health organizations study, it was difficult to draw a conclusion. No contradictions were detected between the studies’ results and policies.

Research conducted in DHRC influenced policies mainly in two ways: (i) a supportive way, where there was already an international policy and the research findings supported it, and (ii) widening accepted beliefs regarding a practice. The level of influence was mainly at national level (four of the six projects).

## Discussion

The results found on the impact of the research conducted in DHRC on national and local policies are linked with several factors that influence policy making- some identified and reported elsewhere while others were identified in this study as described below.

DHRC has been successful in contacting and involving policy makers since research conception. The proximity of the research centre to Accra could have been a facilitating factor. Alternation of the same individuals between research and policy making may also improve the interaction and the translation of research into policies and can be seen in Ghana and in other low and middle income countries [27]. Collaboration and partnership between both communities from research conception until dissemination of findings has been also identified in 5 reviews [28, 29] as a facilitator factor for the use of research for policy making. It helps to address real research needs and increases the willingness to use research results.

The general opinion among policy makers interviewed was that DHRC is successfully meeting research needs. Interviewees believed that addressing health priorities is one of the research determinants for policy impact, which was also identified in 3 reviews [30–32]. Studies belonging to a multi-country initiative (e.g. ACT consortium) and with recognized actors were perceived to have a stronger impact on health policies than studies without this kind of support, as reported elsewhere [27]. Studies with a smaller budget had more influence at the local level than at the national level and its impact on policies was more difficult to determine, although this was not identified in any of the 5 reviews.

Structured communication channels are important to make the research accessible and to promote good governance and less individual or particular influence of specific projects on policies [28]. Interviewees described that researchers used more formal than informal mechanisms. In Ghana, researchers communicate their results during the annual reviews among other ways of disseminating results. Even though the review meetings are part of the policy process, researchers and policy makers from the GHS identify these meetings as a structured way of communicating results to policy makers. These are in fact windows of opportunity in which research results can be influential, and they are also occasions to identify and share health problems. In terms of research products and how DHRC disseminated its results, interestingly there was no direct link between production of articles and inclusion in policies. For example, findings of the “*rectal artesunate study”* study were included in three documents although no article had been produced at that time. Policy briefs were done in only one study, showing that this was not a common practice in DHRC and it was not one of the determinants for policy impact among the studies analysed, contrary to what was reported elsewhere [25]. National dissemination of results (formal or informal) was done in five of the six projects, while international dissemination was done in three studies.

With regards to the impact framework used, authors felt that the RIF was useful and easy to use. However, results might be influenced by the knowledge and vision of the researchers and policy makers interviewed, which suggested possible areas and policy documents that might have been influenced by the research. Although policy makers in general don’t want to speak negatively about their researcher colleagues, the fact that for each study a researcher and a policy maker was interviewed made the process more comprehensive, making it possible to analyse one issue from the two perspectives. Finally, the review of the suggested policy documents to verify the inclusion of research findings was critical to make the evaluation valid. This review also had some challenges as not all documents included references. In those cases, one can only state that the policy is coherent with research findings without ensuring that a particular study influenced a policy.

Results from this study have an importance at different levels. At DHRC level, results can be used to show stakeholders the impact of the research conducted, being accountable and justifying the need for continuous support. It also showed a path to improve DHRC performance. This study sets a practical example on how to evaluate research impact that other research institutions in Ghana and elsewhere can follow. The methodology and results of this study was presented to the other two health research centres and major policy makers of the Ghana Health Service in a seminar on research translation into policy. Although this study did not explore the impact of DHRC at international level, the participation of researchers in international networks and the funding from WHO and the ACT consortium suggest that the research conducted in DHRC might have influenced international policy. Finally, the fact that factors associated to research translation reported in this study were also found in other studies conducted elsewhere, support the validity of our results and reminds what any researcher must consider if he/she aims to influence policy.

## Conclusions

DHRC had policy impact in terms of participation in policy networks, increasing capital value of the researchers and influencing policy documents. DHRC good practices that may explain these positive results included the collaboration with policy makers at the design stage, addressing health priorities, and communicating results mainly through the participation in annual review meetings.

Moving towards continuous improvement, DHRC could consider some recommendations for the next strategic plan to improve the research process and its impact. Some of these recommendations are already described in the strategic plan and they are now being reinforced. Recommendations include conducting research coherent with research agendas and facilitating research collaboration with other research institutions to bring expertise and to increase credibility and power to influence policies. DHRC was already contacting policy makers at the design stage. The findings of this study are encouraging in this respect, and suggest to continue this contact during implementation and dissemination of results. New recommendations are to strengthen the communication of findings through the creation of incentives for publishing, the elaboration of policy briefs, budget lines for communication in each research study and the promotion of staff training on research communication. Another recommendation is to support the district data analysis, setting a technical collaboration between both institutions where district staff can benefit from the scientists’ knowledge on analyses and the scientist can better identify research needs. Finally, DHRC could schedule and conduct policy impact evaluations periodically.

## Additional file

Additional file 1: Table S1. Individually randomized trial of rapid diagnostic tests in rural Ghana. **Table S2.** Home management of fevers in children under-five: a cluster randomized controlled trial in southern Ghana. **Table S3.** Assessments of male involvement in family planning decision making and practice and its influence on the uptake of family planning in the Dangme West district. **Table S4.** Examination of the TB enablers package in the Dodowa sub-district of the Dangme West district in the Greater Accra region of Ghana. **Table S5.** Mutual health organizations (MHO’s) in Ghana and implications for improving the success of health insurance in Ghana. (ODT 59.6 kb)
