# Supplementary material for: Assessing the impact of health research on health policies: a study of the Dodowa Health Research Centre, Ghana
Source: BMC Health Serv Res. 2017 Jun 24;17:435. doi: 10.1186/s12913-017-2383-0 (PMC5482934; doi:10.1186/s12913-017-2383-0)
Supplement: Supplementary file 1 [file 12913_2017_2383_MOESM1_ESM.odt]

| Additional Table 1. Individually randomized trial of rapid diagnostic tests in rural Ghana |
| --- |
| Analysis areas | Key topics | Key dates |
| Research project focus and funding | Research aim: the project aimed to study the impact of RDT on prescribing behaviour of clinicians in two types of health facility settings serving the same population: one where microscopy is routinely available and the other where diagnosis is mainly presumptive. It also explored perceptions of clinicians and patients on the use of RDT in the two settings and its cost-effectiveness. | 2007 |
| Geopolitical context: Dangme West District, Ghana. |  |
| Funders and funding process: Bill and Melinda Gates foundation through the Gates Malaria Partnership (GMP) and the ACT Consortium based at LSHTM. Budget: 177,500US$ |  |
| Research project evolution/ process | Conception: The initiative came from the researcher. The national malaria control program (NMCP) was contacted at this stage. Justification: Ghana changed the first line drug from cloroquine to an ACT. Given that ACTs though highly effective, are substantially more expensive than the previous antimalarial, the economic implications of over diagnosis are considerable. RDTs could improve targeting of malaria treatment and could be cost-effective if clinicians prescribe based on test results. There was also the need to understand how best to support the introduction of RDTs for the management of febrile illness, understanding both clinician and patient’s perspectives of the tests. The study is considered by the researcher and policy maker as addressing an important, urgent and complex issue. |  |
| Study design/Research methods: Randomized controlled trial. The design was defined as good quality study. |  |
| Research collaborators: GMP, LSHTM, NMCP, the district research team, DHRC and ACT consortium for the qualitative work. |  |
| Key project events/concerns: Nothing to mention. |  |
| Main findings/recommendations: Where microscopy already exists, introducing RDT had limited impact on prescriber behaviour. In settings without microscopy, using RDT led to a significant reduction in the over prescription of antimalarials, without any evidence of clinical harm, and to a better targeting of antibiotics. The researcher and the policy maker believed that results were clear and concrete. Dissemination of findings: results were presented to the community, the district authorities, and the regional health management review. Project report was sent to the national level (NMCP). Presentation at international level-American Society of Tropical Medicine & Hygiene Annual Conference. The researcher believed the results have been communicated effectively but more could have been done (a policy brief). |  |
| Research products | Project report and Power point presentation. Done. No policy brief. | 2010 |
| Articles: Four articles were published [[18](#_ENREF_18), [21](#_ENREF_21), [22](#_ENREF_22), [39](#_ENREF_39)]. |  |
| Policy impacts | Level of policy making: National and international level. |  |
| Type of policy impact: clinical practice |  |
| Nature of policy: supportive way: there was an international movement in this direction being done by the ACT Consortium. |  |
| Policy networks: The researcher was a member of a Task Force to develop guidelines for laboratory diagnosis of malaria and participated in the training of trainers. Also, she participated in the ACT consortium. |  |
| Political capital: The researcher participation in the task force helped to value the research evidence as well as the researcher. |  |
| Policy discourse and debate: there were discussions about the need of rationalization on the use of antimalarial. Hospitals with microscopy wanted to use the RDT even though the guidelines indicated that they should be used in health facilities where microscopy was unavailable. The NMCP compromise on this was to use the RDTs when the laboratory was not functioning or laboratory technicians were unavailable. |  |
| Inclusion in policy documents: National guidelines for laboratory diagnosis for malaria, and guidelines for case management of malaria. Who benefited: Community and the health system in terms of cost effectiveness of interventions. Unexpected/intended outcomes: malaria prevalence was less than expected. | 2008 2009 |

| Additional Table 2. Home management of fevers in children under-five: a cluster randomized controlled trial in southern Ghana |
| --- |
| Analysis areas | Key topics | Key dates |
| Research project focus and funding | Research aim: to evaluate the impact of adding an antibiotic (amoxicillin) to an antimalarial (artesunate amodiaquine) for treating fever among children 2-59 months of age within the HBC strategy. |  |
| Geopolitical context: Dangme West district, Ghana |  |
| Funders and funding process: WHO call | 2007-2009 |
| Budget: 856,000$ |  |
| Research project evolution/ process | Conception: Researchers from DHRC applied to the call.  Justification: Under five mortality increased in Ghana from 1999 to 2003 and remained stagnant in 2006. Main causes of death were malaria (16%) and diarrhoea (15%). This research wanted to see if adding an antibiotic to an antimalarial in the HBC strategy could reduce child mortality more effectively. |  |
| Study design/Research methods: Cluster randomized control trial. The study is considered by the researcher and policy makers as addressing an important health problem, an urgent matter and a complex issue. The design was defined as good quality study although they used historical controls due to money and time limitations. |  |
| Research collaborators: WHO, Makerere university (Uganda), GHS (malaria and child health programs), DHRC, university of Amsterdam, university of Maastricht. |  |
| Key project events/concerns: During the project implementation, RDT came into the picture, giving an opportunity to distinguish between malaria and pneumonia. |  |
| Main findings/recommendations: mortality reduced by 30% in those using antimalarial and by 44% in those using antimalarial and antibiotic, compared with the standard care. The reduction of mortality between antimalarial alone or adding an antibiotic was not statistically significant. These findings are considered to be replicable in similar settings. |  |
| Dissemination of findings: to the community, to WHO and at the Global Health Forum in Geneva in 2008: “Development of a Strategy for Home and Community Management of Malaria and Pneumonia in Children Under Five in Southern Ghana”. To other stakeholders only informally. To other stakeholders only informally. No presentation at the dissemination forum (forum was postponed). |  |
| Main research products | Project report and Power point presentation. Done. No policy brief. |  |
| Articles: Two articles were published [[19](#_ENREF_19), [20](#_ENREF_20)]. |  |
| Policy impacts | Level of policy making: the study added more evidence on the efficacy of malaria home base care in Ghana that was already being implemented. |  |
| Type of policy impact: The level of influence is at national level, on service policies and clinical practice policies. |  |
| Nature of policy: research evidence supported the existing policies on HBC. |  |
| Policy networks: authors belong and have links with INDEPTH, TDR/WHO and iCCM task force. The project contributed to increase those links. |  |
| Political capital: The researcher believed the project increased the respect and power of authors on policy dialog. |  |
| Policy discourse and debate: how to better manage fever at community level, reducing child mortality being cost effective. RDT are not included yet on the HBC, so adding an antibiotic could have been an option at community level. |  |
| Inclusion in policy documents: research results supported the HBC strategy in Ghana. The malaria, diarrhoea and pneumonia home based care guidelines prescribes amoxicillin only when there are respiratory signs of pneumonia. Who benefited: the community and the health system level, having community based agents trained for the HBC strategy. Unintended outcomes: None | 2010 |

| Additional Table 3. Assessments of male involvement in family planning decision making and practice and its influence on the uptake of family planning in the Dangme West district. |
| --- |
| Analysis areas | Key topics | Key dates |
| Research project focus and funding | Research aim: the project aimed to assess the extent and nature of male involvement in FP in order to increase family planning utilization. | 2005 |
| Geopolitical context: Dangme West District, Ghana. |  |
| Funders and funding process: Ghana- Dutch collaboration |  |
| Budget: 5.400$ |  |
| Research project evolution/ process | Conception: The Ghana- Dutch collaboration helped the GHS to develop research priorities and to finance some projects. This topic is one of the areas the family division works on: repositioning male involvement on FP. Justification: The study is considered by the researcher and policy makers as addressing an important health problem, an urgent matter (except for 1 policy maker) and a complex issue. |  |
| Study design/Research methods: Cross sectional study (household survey) and Qualitative methods (focus groups discussions, in-depth interviews and case studies). The design was defined as good quality study by the researcher and policy makers. |  |
| Research collaborators: Dutch government and district team with its health facilities. Those collaborators were involved in the study from the conception until the dissemination of results. The Ghana-Dutch collaboration gave financial and technical support. |  |
| Key project events/concerns: the project was identified to address a district program challenge. No national impact was expected. |  |
| Main findings/recommendations: the study produced evidence that men are not generally involved in FP while they are the decision makers. Reasons for not accepting FP were perception on manhood and women promiscuity. Some recommendations made where inclusion of man health staff in FP services, and sensitization of men and social groups on FP. Results are considered to be enough clear and concrete and replicable in other settings. |  |
| Dissemination of findings: results were presented to the district authorities and at the research dissemination forum reaching around 100 people. A study report was included in the DHRC annual report. The researcher believes that the results could have been communicated more effectively, especially at national level, for example communicating directly the results to family planning division. |  |
| Main research products | Project report and Power point presentation: Done | 2006 |
| Articles: no article has been produced. |  |
| Policy impacts | Level of policy making: Researcher expressed that if there was an impact at district level that was due to different studies done at the same time and not exclusively to that one. However, the former district director explained that after that study, health education sessions were organized in the community, inviting males to eliminate misconceptions. The use of FP has increased in the district (from 8.7% in 2007 to 47% in 2011), mainly due to community campaigns conducted in 2009 and 2010. |  |
| Type of policy impact: the study influenced service policies on the use of FP. |  |
| Nature of policy: redefining/wider the influence in accepted beliefs and practices. |  |
| Policy networks: The researcher believed that no impact on policy networks was achieved due to the project. The PM thinks it had an impact at district level. |  |
| Political capital: researcher believes that no impact on political capital was achieved due to the project. |  |
| Policy discourse and debate: At the point of the study, there was a national concern on the low utilization of FP methods. Low male involvement could be one of the reasons for the low utilization and needed to be addressed. |  |
| Inclusion in policy documents: the 2009 and 2010 district annual reports showed realization of more extensive community campaigns on FP. Who benefited: Directly, men and women in the communities of Dangme West district. Indirectly, their children as well as the research capacity. Unintended outcomes: None | 2009 2010 |

| Additional Table 4. Examination of the TB enablers package in the Dodowa sub-district of the Dangme West district in the Greater Accra region of Ghana |
| --- |
| Analysis areas | Key topics | Key dates |
| Research project focus and funding | Research aim: the project aimed to explore the effectiveness of the enabler’s package in Dodowa, looking at the provision, accessibility and patient’s usage. | 2009 |
| Geopolitical context: Dangme West District, Ghana. |  |
| Funders and funding process: George Town University. |  |
| Budget: 6,000$ |  |
| Research project evolution/ process | Conception: The initiative came from the district and research centre, in coherence with the research agenda set with the support of the Ghana- Dutch collaboration. The TB national control program encourages districts to conduct their own research. The strategic plan 2009-2013 specifies that one of the activities is to conduct a situation analysis on enablers. Justification: The TB program received complains about TB package abuse. TB enablers is one of the core activities of the program. The study is considered by the researcher and policy makers as addressing an important health problem, an urgent matter and a complex issue. |  |
| Study design/Research methods: Cross sectional study, with qualitative and quantitative data. The design was defined as good quality study. |  |
| Research collaborators: District team and DHRC. |  |
| Key project events/concerns: small number of cases (12 cases in the district). |  |
| Main findings/recommendations: 50% of cases interviewed did not receive any package. Home visits were irregular and the involvement of the community in the program was low. No recommendations made. Findings cannot be generalized to other districts. |  |
| Dissemination of findings: Dissemination was local. Informal communication to the TB program. | 2010 |
| Main research products | Project report and Power point presentation: Done. No policy brief |  |
| Articles: None |  |
| Policy impacts | Level of policy making: mainly at district level, affecting heath managers and providers. |  |
| Type of policy impact: governance of the intervention, the way the enabler’s package has to be distributed and supervised. |  |
| Nature of policy: Redefining and widening influence: research leads to a change in current practices of TB enablers. |  |
| Policy networks: Participation in the local dissemination mechanism. |  |
| Political capital: the study increased the relationship between the research centre and the program, resulting in a new research collaboration 2011. |  |
| Policy discourse and debate: There was a need to evaluate the TB package in the country. |  |
| Inclusion in policy documents: The 2011 district annual report described for the first time who benefited from the enablers package, suggesting some influence from the project.  Who benefited: district managers and TB patients. It helped to verify miss use of enablers and the need to improve supervision. Unexpected/intended outcomes: None | 2011 |

| Additional Table 5. Mutual health organizations (MHO's) in Ghana and implications for improving the success of health insurance in Ghana |
| --- |
| Analysis areas | Key topics | Key dates |
| Research project focus and funding | Research aim: to prepare an inventory of risks mechanisms and other technical features of mutual health schemes in Ghana and its effects on economic sustainability of schemes. |  |
| Geopolitical context: Ghana |  |
| Funders and funding process: Ghana- Dutch collaboration |  |
| Budget: 6,081$ | 2004 |
| Research project evolution/ process | Conception: The initiative came from researchers, after a previous study on community satisfaction, accessibility and sustainability of Dangme West mutual scheme. Justification: The National Health Insurance Scheme (NHIS) was about to start. Experience from existing MHO’s may benefit the new NHIS. The study is considered by the PI and Policy makers as addressing an important health problem, an urgent matter and a complex issue. |  |
| Study design/Research methods: review of the existing MHO’s looking at the structure, financial management and financial position of schemes. The design was defined as good quality study. |  |
| Research collaborators: DHRC, Erasmus University (iMTA), district health research team, research division (GHS), and Dutch government. MHO’s were contacted as well during the study design. |  |
| Key project events/concerns: difficult communication in the whole country (roads and phone), inclusion criteria, mistrust of MHO (due to political issues). |  |
| Main findings/recommendations: Private schemes had more autonomy in setting premiums and benefit package, with higher community participation. Schemes had few measures to control moral hazard (mainly though referral letters) and adverse selection (mainly though encouraging household registration) but more to control fraud and cost escalation. Recommendations: regulation of schemes, building trust and allowing enough autonomy to adapt to local situations. Results are considered to be clear and concrete. |  |
| Dissemination of findings: 4 presentations: at University of Ghana (SPH), at Dissemination forum, Public dissemination and at Erasmus University. Not dissemination to participants due to limited money. More than 300 hundred people received the research results at the end of the study. |  |
| Main research products | Project report and Power point presentation. Done. Policy brief was done and sent to the GHS (Policy, Planning, Monitoring and Evaluation). | 2006 |
| Articles: One article was published [[40](#_ENREF_40)]. |  |
| Policy impacts | Level of policy making: At national level, at provider’s level. |  |
| Type of policy impact:  On governance policies |  |
| Nature of policy: instrumental use of the research: the knowledge of the PI was used to help the design and implementation of the NHIS. |  |
| Policy networks: One of the authors belongs to a team created to roll out the national insurance. |  |
| Political capital: one of the authors is a reference person on NHIS in Ghana. |  |
| Policy discourse and debate: At this stage, the NHIS was starting. The NHIS dates from 2003, the regulation from 2004 and the implementation from 2005. |  |
| Inclusion in policy documents: The NHIS act was created in 2003 and its regulation in 2004. Real implementation started in 2005. Interviewees could not show policy documents were research results were included. Who benefited: academics, policy makers, politicians (NPP as initiating NHIS), NGOs (advocating for the NHIS), CHAG (advocating for access for the poor) and community members. Unintended outcomes: None |  |

Additional file. Questionnaires

| London School of Hygiene & Tropical Medicine (University of London) Keppel Street, London WC1E 7HT |  |
| --- | --- |

Semi-structure interview to Policy makers related to the projects selected

Name of Policy Maker: ___________________________________________________

Actual position Policy maker holds at present: ________________________________

| Introduction |
| --- |

Are you familiar with the Project we are going to talk about? If yes, continue with the interview.

If not, make a summary of the Project and ask for policy documents related to the study to analyse if research results are in coherence with the policy document. Then, change the questionnaire and use the one for policy makers “NO related to the projects selected”.

Research project conception

- How this research Project was identified/ suggested? Where did the initiative come from? Please explain.

- Which institution/persons were involved during the identification/conception of this research Project? Please explain.

- In your institution/department, do you have budget to do research?

- Does your institution/department have a research agenda to guide research? How do you identify a need for research?

- Base on your experience, how health research is more often identified/ suggested in your institution/department? Where did the initiative come from?

- Which institutions/departments do you normally contact if do you think there is a need for research. Please explain. Do research groups/institution contact you when identifying research?

- What is the way your institution interacts with research groups? How do you see your role and the research group role in this interaction?

- What barriers or challenges, if any, have you experienced in working with research in terms of research conception? Please explain any barriers/challenges and how they were addressed (or could have been addressed).

Research project design

- Is this research project addressing an important health problem? Why? (Please explain).

- Is this research project addressing an urgent matter? Why? (Please explain)

- Is this research project addressing a complex issue? Why? (Please explain)

- Are the research results replicable in other settings? Why? (Please explain)

- How would you qualify the methodology of this research project, in terms of quality? Why? (Please explain)

- Which institution/persons were involved during the project design? Please explain

- Have you received assistance from other institutions, national or international (e.g., financial, educational, technical, products, services) during the project design? Please describe the assistance received and comment on its effectiveness.

- What barriers or challenges, if any, have you experienced in working with research in terms of research design? Please explain any barriers/challenges and how they were addressed (or could have been addressed).

Research project implementation

- Is this research part of a multi country study? Please explain

- Do you know which institution/persons were involved during the implementation? Please explain

- Do you know if the Project has received assistance from other institutions, national or international (e.g., financial, educational, technical, products, services) during the project implementation? Please describe the assistance received and comment on its effectiveness.

- What barriers or challenges, if any, have you experienced in working with the Project in terms of research implementation, (data collection, analysis and writing)? Please explain any barriers/challenges and how they were addressed.

Communication and Dissemination of research project results

- Do you think the results recommendations were clear and concrete? Please explain.

- Which source of information do you normally use to look for evidence to justify/inform policy/practice decisions? Please explain

- In that Project, do you think researchers have communicated effectively research results to you and to other key policy makers? Please explain.

- How have you received information about research results of this Project?

- Please estimate at which point of the study you have received/discussed research results about the Project. 

- What barriers or challenges, if any, have you experienced in working with the Project in terms of research communication/ dissemination? Please explain any barriers/challenges and how they were addressed.

| Research project Policy Impact |
| --- |

Based on your project involvement, knowledge, or expertise, in which areas below has the Project had a policy impact? Please provide evidence for the areas for which you indicate the Project has had an impact.

The Impact Framework[^1] below provides an overview of possible Project policy impacts.  The Project may or may not have had an impact in all areas.  Please read through each impact category and subcategory as there may be areas you have not considered previously.

| Subcategories of Policy Impact | Examples of Potential Impacts | Answer | Stakeholder Provided Evidence of Project Impact |
| --- | --- | --- | --- |
| At which level of policy making did the project have an impact? | policy impacts at international, national, state, and local levels; policy impacts on a range of stakeholders such as legislators, health managers and providers, health-related organizations, associations, and agencies, academics, communities or community-based organizations | [ ] A [ ] B [ ] C [ ] No impact [ ] Don’t Know Please explain |  |
| Which type of policy impact did your project have? | clinical practice policies (ACT for malaria) service policies (health promotion in primary care), governance policies (reorganization of NHIS); | [ ] A [ ] B [ ] C [ ] No impact [ ] Don’t Know Please explain |  |
| Which nature of policy impact did your project have? | instrumental use: research findings directly drive or define policy; mobilization of support where research findings provide supportive evidence to back policy activities and or raise awareness of and support for policy-making; conceptual use where research leads to new ideas and language influencing policy discourse; redefining/wider influence whereby research leads to a wide change in accepted beliefs and practices | [ ] A [ ] B [ ] C [ ] D [ ] No impact [ ] Don’t Know Please explain |  |
| In which type of Policy networks did your project have an impact? | e.g., extent to which researchers are a part of, or inform, policy networks through, for instance, policy advocacy groups, think tanks and government institutions | [ ] Yes. [ ] No impact [ ] Don’t Know Please explain and list collaborations if any____________________ |  |
| Did you project have an impact on Political capital? | e.g., value of research evidence and researchers themselves in policy negotiations, reaching quality agreements and improvements in the policy-making process, in ability in societies to achieve agreed-upon ends, and in socio-political prestige resulting from innovations | [ ] Yes. [ ] No [ ] Don’t Know Please explain |  |

- Which stakeholders (including target populations) have benefited from the activities of the Project so far? Please explain

- What is the Project’s greatest research benefit to you, your organization, or other stakeholders?

- What unexpected or unintended outcomes (positive or negative) have you seen from this Project?

- What barriers or challenges, if any, have you experienced in working with the Project in terms of using this research results for policy making? Please explain any barriers/challenges and how they were addressed.

- In general terms now and based on your expertise, which type of research do you paid more attention (related to clinical practice, related to health system level, related to Behavioural level,)? 

- Based on your expertise, which factors are more related to the quality of the research? Please explain

- Based on your expertise, which factors related to the content of the research are more important and will make policy makers more interested in the research results? Which factors are not? Please explain

- Based on your expertise, which factors related to the actors involved in the research/topic are more important and will make policy makers more interested in the research results? Which factors are not? Please explain.

- Based on your expertise, which factors related to the context of the research/policy process may affect the relationship between researchers and policy makers and the use of the research? Which factors may not? Please explain.

- Is there any norm/law about research that can be relevant for this study on policy impact?

- Do you recommend me to interview another stakeholder that can provide more information about the policy impact of this project?

| London School of Hygiene & Tropical Medicine (University of London) Keppel Street, London WC1E 7HT |  |
| --- | --- |

Semi-structure interview to Policy makers No related to the projects selected

Name of Policy Maker: ___________________________________________________

Position Policy maker holds at present: ___________________________________

| Introduction |
| --- |

As you were not involved in the projects selected, I am going to ask you general questions about how you, your department and your institution deals with research conception, design, dissemination of results and use of evidence for policy making.

| Research project conception |
| --- |

1. In your institution/department, do you have budget to do research?

2. Does your institution/department have a research agenda to guide research? How do you identify a need for research?

3. Base on your experience, how health research is more often identified/ suggested in your institution/department? Where did the initiative come from?

4. Which institutions/departments do you normally contact if you think there is a need for research? Please explain. Do research groups/institution contact you when identifying research?

5. What is the way your institution interacts with research groups? How do you see your role and the research group role in this interaction?

6. What barriers or challenges, if any, have you experienced in terms of research conception? Please explain any barriers/challenges and how they were (or could be) addressed.

Research project design and content

Dodowa Health research centre (DHRC) has existed since early 1990. Five DHRC projects have been selected to look at the impact of health research on health policies based on selected criteria:

- Mutual Health Organizations (MHO’s) in Ghana and implications for improving the success of health Insurance in Ghana.

- Assessments of male involvement in family planning decision making and practice and its influence on the uptake of Family Planning in the Dangme West district.

- Deployment of rectal articulate in the Dangme West district for severe malaria in children under five.

- Home management of fevers (malaria and pneumonia) in children under-five: a cluster randomized controlled trial in southern Ghana.

- Examination of the TB Enablers Package in the Dodowa sub-district of the Dangme West District in the Greater Accra Region of Ghana.

- Individually randomized trial of rapid diagnostic tests in rural Ghana 

- Based on the titles – Do you think these research projects address important health problems? Why? (Please explain).

- Do you think that these research projects address an urgent matter? Why? (Please explain)

- Do you think that these research projects address a complex issue? Why? (Please explain)

- What barriers or challenges, if any, have you experienced in terms of research design? Please explain any barriers/challenges and how they were or could be addressed.

Communication and Dissemination of research project results

- How do you get to know about the research done in Ghana, and more specifically in DHRC? Is there a defined mechanism to receive research results from Ghanaian research institutions/centres? Please explain 

- Which sources of information do you normally use to justify/inform policy/practice decisions? 

- What barriers or challenges, if any, have you experienced in terms of research communication/ dissemination? Please explain any barriers/challenges and how they were or could be addressed.

| Research project Policy Impact |
| --- |

- Based on your expertise, which type of research do you paid more attention (related to clinical practice, related to health system level, related to Behavioural level,)? 

- Based on your expertise, which factors are more related to the quality of the research.

- Based on your expertise, which factors related to the content of the research are more important and will make policy makers more interested in the research results? Which factors are not? Please explain

- Based on your expertise, which factors related to the actors involved in the research/topic are more important and will make policy makers more interested in the research results? Which factors are not? Please explain.

- Based on your expertise, which factors related to the context of the research/policy process may affect the relationship between researchers and policy makers and the use of the research? Which factors may not? Please explain.

- Is there any norm/law about research that can be relevant for this study?

- What barriers or challenges, if any, have you experienced in terms of research impact? Please explain any barriers/challenges and how they were addressed.

- Do you recommend me to interview another stakeholder that can provide more information about the research and policy impact?

| London School of Hygiene & Tropical Medicine (University of London) Keppel Street, London WC1E 7HT |  |
| --- | --- |

Semi-structure interview to PI

Name of the Project: __________________________________________________________

Name of PI: ___________________________________________________________

Research project conception

- How this research Project was identified/ suggested? Where did the initiative come from? Please explain.

- Which institution/persons were involved during the identification/conception of this research Project? Please explain.

- Base on your experience, how health research is more often identified/ suggested in your institution/department? Where did the initiative usually come from?

- Which institutions/departments do you normally contact if you think there is a need for research? Please explain. 

- What is the way your institution interacts with policy makers? How do you see your role and the policy maker’s role in this interaction?

- What barriers or challenges, if any, have you experienced in terms of research conception? Please explain any barriers/challenges and how they were addressed (or could have been addressed).

Research project design

- Is this research project addressing an important health problem? Why? (Please explain).

- Is this research project addressing an urgent matter? Why? (Please explain)

- Is this research project addressing a complex issue? Why? (Please explain)

- Are the research results replicable in other settings? Why? (Please explain)

- How would you qualify the methodology of this research Project, in terms of quality? Why? (Please explain)

- Which institution/persons were involved during the project design? Please explain

- Have you received assistance from other institutions, national or international (e.g., financial, educational, technical, products, services) during the project design? Please describe the assistance received and comment on its effectiveness.

- What barriers or challenges, if any, have you experienced in terms of project design? Please explain any barriers/challenges and how they were addressed (or could have been addressed).

Research project implementation

- Is this research part of a multi country study? Please explain

- Which institution/persons were involved during the implementation? Please explain

- Was the money of the project enough for an adequate implementation? Please explain

- Have you received assistance from other institutions, national or international (e.g., financial, educational, technical, products, services) during the project implementation? Please describe the assistance received and comment on its effectiveness.

- What barriers or challenges, if any, have you experienced in working with the Project in terms of research implementation, (data collection, analysis and writing)? Please explain any barriers/challenges and how they were addressed (or could have been addressed).

Communication and Dissemination of research project results

- Do you think the results recommendations were clear and concrete? Please explain 

- Which way of communication do you normally use to disseminate research results to policy makers? Please explain

- In that Project, do you think researchers have communicated effectively research results to key policy makers? Please explain.

- How have you shared information with stakeholders (including target populations) about the Project and its results?

- Please estimate at which point of the study you have shared information/discussed about the Project with policy makers. 

- Please estimate the number of people with whom you have shared information about the Project. 

- What barriers or challenges, if any, have you experienced in working with the Project in terms of research communication/dissemination? Please explain any barriers/challenges and how they were addressed (or could have been addressed).

| Research project Policy Impact |
| --- |

Based on your project involvement, knowledge, or expertise, in which areas below has the Project had a policy impact? Please provide evidence for the areas for which you indicate the Project has had an impact.

The Impact Framework[^2] below provides an overview of possible Project policy impacts.  The Project may or may not have had an impact in all areas.  Please read through each impact category and subcategory as there may be areas you have not considered previously.

| Subcategories of Policy Impact | Examples of Potential Impacts | Answer | Stakeholder Provided Evidence of Project A Impact |
| --- | --- | --- | --- |
| At which level of policy making did the project have an impact? | policy impacts at international, national, state, and local levels; policy impacts on a range of stakeholders such as legislators, health managers and providers, health-related organizations, associations, and agencies, academics, communities or community-based organizations | [ ] A [ ] B [ ] C [ ] No impact [ ] Don’t Know Please explain |  |
| Which type of policy impact did your project have? | clinical practice policies (ACT for malaria) service policies (health promotion in primary care), governance policies (reorganization of NHIS); | [ ] A [ ] B [ ] C [ ] No impact [ ] Don’t Know Please explain |  |
| Which nature of policy impact did your project have? | instrumental use: research findings directly drive or define policy; mobilization of support where research findings provide supportive evidence to back policy activities and or raise awareness of and support for policy-making; conceptual use where research leads to new ideas and language influencing policy discourse; redefining/wider influence whereby research leads to a wide change in accepted beliefs and practices | [ ] A [ ] B [ ] C [ ] D [ ] No impact [ ] Don’t Know Please explain |  |
| In which type of Policy networks did your project have an impact? | e.g., extent to which researchers are a part of, or inform, policy networks through, for instance, policy advocacy groups, think tanks and government institutions | [ ] Yes. [ ] No impact [ ] Don’t Know Please explain and list collaborations if any |  |
| Did your project have an impact on Political capital? | e.g., value of research evidence and researchers themselves in policy negotiations, reaching quality agreements and improvements in the policy-making process, in ability in societies to achieve agreed-upon ends, and in socio-political prestige resulting from innovations | [ ] Yes. [ ] No [ ] Don’t Know Please explain |  |

- Which stakeholders (including target populations) have benefited from the activities of the Project so far? Please explain

- What is the Project’s greatest research benefit to you, your organization, or other stakeholders?

- What unexpected or unintended outcomes (positive or negative) have you seen from this Project?

- What barriers or challenges, if any, have you experienced in working with the Project in terms of using this research results for policy making? Please explain any barriers/challenges and how they were addressed.

- In general terms now, and based on your expertise, which type of research do you think policy makers paid more attention (related to clinical practice, to health system level, to Behavioural level,)? 

- Based on your expertise, which factors are more related to the quality of the research? Please explain

- Based on your expertise, which factors related to the content of the research are more important and will make policy makers more interested in the research results? Which factors are not? Please explain

- Based on your expertise, which factors related to the actors involved in the research/topic are more important and will make policy makers more interested in the research results? Which factors are not? Please explain.

- Based on your expertise, which factors related to the context of the research/policy process may affect the relationship between researchers and policy makers and the use of the research? Which factors may not? Please explain.

- Is there any norm/law about research that can be relevant for this study on policy impact?

- Do you recommend me to interview another stakeholder that can provide more information about the policy impact of this project?

[^1]: Kuruvilla, S., Mays, N., Pleasant, A., and Walt, G.  (2006). describing the impact of health research:  A Research Impact Framework.  BMC Health Services Research, 6:134, doi:  10.1186/1472-6963-6-134.  Article available from http://www.biomedcentral.com/1472-6963/6/134
[^2]: Kuruvilla, S., Mays, N., Pleasant, A., and Walt, G.  (2006). describing the impact of health research:  A Research Impact Framework.  BMC Health Services Research, 6:134, doi:  10.1186/1472-6963-6-134.  Article available from http://www.biomedcentral.com/1472-6963/6/134.
